# Surface Roughness Model Based on Force Sensors for the Prediction of the Tool Wear

**DOI:** 10.3390/s140406393

**Published:** 2014-04-04

**Authors:** Beatriz de Agustina, Eva María Rubio, Miguel Ángel Sebastián

**Affiliations:** Department of Manufacturing Engineering, Industrial Engineering School, National University of Distance Education (UNED), C/Juan del Rosal, 12, E28040-Madrid, Spain; E-Mails: erubio@ind.uned.es (E.M.R.); msebastian@ind.uned.es (M.Á.S.)

**Keywords:** dry turning, aluminum alloys, force sensor, surface roughness models, tool wear, design of experiments, ANOVA, regression

## Abstract

In this study, a methodology has been developed with the objective of evaluating the surface roughness obtained during turning processes by measuring the signals detected by a force sensor under the same cutting conditions. In this way, the surface quality achieved along the process is correlated to several parameters of the cutting forces (thrust forces, feed forces and cutting forces), so the effect that the tool wear causes on the surface roughness is evaluated. In a first step, the best cutting conditions (cutting parameters and radius of tool) for a certain quality surface requirement were found for pieces of UNS A97075. Next, with this selection a model of surface roughness based on the cutting forces was developed for different states of wear that simulate the behaviour of the tool throughout its life. The validation of this model reveals that it was effective for approximately 70% of the surface roughness values obtained.

## 1. Introduction

The integration of automated systems in machining processes in the absence of an operator requires not only the best selection of cutting parameters, but also the monitoring and control of the process in real time to obtain the level of quality required of the products at a high rate of productivity. In this context, in the last decades a great effort has been made towards the development of monitoring machining systems. The success of such systems is conditioned by their capability of detecting any anomaly during the machining to implement at this point the corresponding corrective actions, maintaining in this way the stability of the process, and avoid downtime of the machine. For this reason, models reported in numerous studies have been developed to simulate the conditions during the machining processes; the parameters extracted from signals detected by different sensors such as dynamometers, acoustic emission, accelerometers, current/power among others, are employed to establish a relationship between several cutting variables and the desired product characteristic, mainly, the surface roughness or the tool wear [1–8].

Up to now the models of surface roughness proposed in previous studies [9–13] have been made by setting different values of the cutting parameters, therefore they show, in most of the cases, a strong relationship dependency between the independent inputs and the desired output (surface roughness).

Nevertheless, due to the complexity of the machining process and the presence of numerous incontrollable factors (tool wear, material workpiece properties and environmental conditions), the implementation of these models in monitoring machining systems is nowadays highly restricted.

In this study, in order to determine, quantitatively, the effect of the tool wear on the surface roughness a methodology has been developed to obtain a model of surface roughness based on the cutting forces under the same cutting conditions to insulate the effect of the tool wear, being, in this sense, novelty with respect to the models mentioned previously. Concretely the aim was to achieve a predictive model of surface roughness by means of different statistic parameters of the cutting forces (thrust forces, feed forces and cutting forces) that could indicate when the surface roughness obtained on pieces by turning is not adequate according to the requested specifications. At this point, it is proposed that the recording of the signals of forces during the machining stops and a visual signal advises the operator.

The methodology developed has been applied in the aeronautical field in which materials such as aluminium alloys are employed for the production of different elements that make up airships and aerospace vehicles due to their combination of properties such as high mechanical resistance, even at high temperatures, as well as a low density. In addition these elements have to meet stringent surface quality requirements. Therefore, an aluminium alloy (UNS A97075), was selected for the development of a model of surface roughness obtained by a dry turning process. In a first step, the most improved cutting conditions (cutting parameters and radius of tool) according to aeronautical surface requirements were found. Secondly, with this selection a model of surface roughness based on the cutting forces at different states of wear was developed that simulates the behaviour of the tool throughout its life.

## 2. Experimental Section

This section includes, firstly, the different stages of the methodology proposed and the objectives pursued in each stage, and secondly, the protocols for the experimental procedure which include the identification of the resources and type of tests, the steps for the acquisition of the measurements (forces and surface roughness) and the statistical tools employed for the analysis of results.

### 2.1. Stages of the Methodology Proposed

The methodology proposed to carry out the experimental research is divided into the following three stages:

#### 2.1.1. First Stage

The first step allows adjustment of the configuration of the registration forces and improvement of the selection of the cutting parameters. For this objective, it is proposed to perform short tests with duration less than 10 seconds under different cutting conditions (cutting parameters and type of tool or material workpiece). During the tests cutting forces must be recorded to analyse their magnitude and their frequency ranges as well as the influence of the different cutting parameters on the forces.

#### 2.1.2. Second Stage

The second step provides, on the one hand, a surface roughness model based on the cutting forces under different cutting conditions and, on the other hand, the best selection of cutting parameters and material workpiece or type of tool according to the surface roughness requirements. Therefore, it is proposed to carry out medium tests employing the time consumed for the machining along the length of the bar in which cutting forces and surface roughness measurements must be taken.

#### 2.1.3. Third Stage

In this stage, a model of surface roughness based on the cutting forces under different states of the tool wear is obtained to simulate the behaviour of the tool during its life. To address this objective, with the combination of the most improved cutting conditions obtained previously, long tests with duration of at least 15 min at different states of tool wear are performed. For each state of tool wear, at different times, cutting forces surface roughness must be taken. Finally, with the aim of measuring and supervising the surface roughness online, it is necessary to implement the model of surface roughness obtained in the cutting forces acquisition system. In addition, according to the surface roughness requirements for a specific application, the allowed limits of the values of surface roughness must also be configured. In such a way that when the value of surface roughness obtained during the process is higher than the imposed limit, a visual or auditory signal indicates the surface roughness measured online is not adequate.

### 2.2. Experimental Protocols

#### 2.2.1. Identification of the Turning Tests and Resources Employed

This investigation is framed within a series of studies in which different materials, types of tools, turning tests, cutting conditions and measurements are involved. In order to systematize the experimental procedure, alphanumeric codes are used to identify all the different parameters and conditions that are considered for analysis. The identification of each turning test consists on the use of alphanumeric codes to indicate the duration of the turning test and the cutting parameters.

The tests are divided mainly into three categories according to the duration of the test in the following way:

#### 2.2.2. Short Tests

For this stage, the identification suggested is ST-FFF-SSSS-DDD being:

ST: Short tests
FFF: Value of the feed rate in mm/rev
SSSS: Value of the spindle speed in revolution per minute (rpm)
DDD: Value of the depth of cut expressed in mm

#### 2.2.3. Medium Tests

The duration of these tests is the time consumed for the machining along the length of the bar employed. The identification for these tests is MT-FFF-SSSS-DDD-L1/L2/..LN-G1/G2..GM, being:

MT: Medium tests.
FFF-SSSS-DDD: The same codes that in the short tests case. That is, values of the feed rate in mm/rev, values of the spindle speed in revolution per minute (rpm) and values of the depth of cut expressed in mm.
L1/L2. LN: L is the longitudinal section of the bar on where the measurement is made and N is the total number of the sections considered (up to three are suggested).
G1/G2. GM: G is the generatrix of the bar on where the measurement is made and M is the total number of generatrix considered (up to three are suggested).

For these types of tests, as surface roughness measurements are made on the workpiece, the last two alphanumeric codes have to be added in order to establish in which part of the workpiece the measurement is carried out.

**Figure 1.**
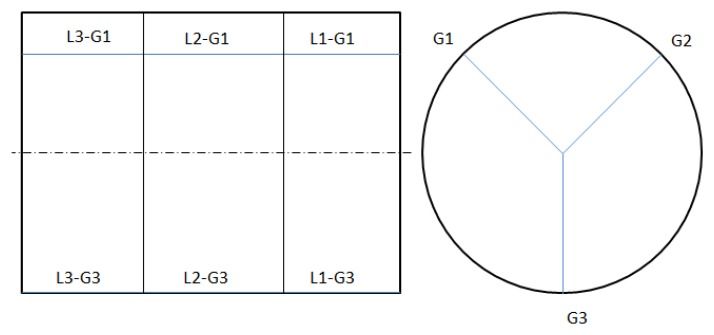
Side and from view of the workpiece.

In Figure 1, a sketch of the workpiece is represented with the locations of the longitudinal sections and generatrix indicated:

#### 2.2.4. Long Tests

The designation proposed for these tests is LT-FFF-SSSS-DDD-W0/W1/W2/WR-T1/T2/TK being:

LT: Long tests.
FFF-SSSS-DDD: The same codes that in the short and medium tests cases. That is, values of the feed rate in mm/rev, values of the spindle speed in revolution per minute (rpm) and values of the depth of cut expressed in mm.
W0/W1/W2/WR: W is the state of wear of the tool that is tested and R the higher level of wear reached (R=0 corresponds to a fresh tool).
T1/T2/TK: T is the time of machining in minutes at that stage (this is, when a surface roughness measurement is carried out) and K is the number of periods that are considered.

In relation to the tools, in spite of the fact there is already a codification for turning inserts collected in the ISO 1832 standard [14], in this study with the aim of simplifying such codification, a new codification is designed to systematically identify the tools in such a way that, on the one hand, the features that are the object of the analysis are considered and, on the other hand, the insert and the edge that is used for a specific turning test are identified.

### 2.3. Previous Activities of the Turning Tests

These activities consist of the configuration of the force sensor amplifier and the corresponding software to register the cutting forces. It is also suggested to carry out one pass of turning with a tool for roughing to prepare the workpiece or workpieces.

### 2.4. Turning Tests

In each test, a workpiece is machined during a certain time of machining under certain conditions of feed rate, cutting speed and depth of cut.

### 2.5. Registration of the Measuring Forces

The cutting forces are recorded and simultaneous graphics are plotted during the tests. From each test, the statistical values from the cutting forces (*F*_x_, *F*_y_ and *F*_z_, corresponding to the feed, thrust and cutting forces, respectively) are calculated. These are their averages (*Fm*_x_, *Fm*_y_, *Fm*_z_) and their standard deviations (*Fd*_x_, *Fd*_y_, *Fd*_z_) in the time domain. In the frequency domain, from the Fast Fourier Transform (*FFT*) peak tracking signal, the three positions of the maximum values in the signals in the three directions (*Pos*_x_, *Pos*_y_ and *Pos*_z_) and the maximum values of these peaks (*Max_x_*, *Max_y_* and *Max_z_*) are calculated.

### 2.6. Previous Activities to the Roughness Measurements

In order to systematize the roughness measurements on the tested pieces, firstly, the measurement process has to be defined. This is, basically, to measure the roughness along two or three generatrix separated by π or 2π/3 radians in each one of the tested pieces. Thus, to carry out the measurements, it is necessary to possess a series of auxiliary elements and measurement instruments and to verify that they are all in perfect state.

### 2.7. Roughness Measurements

According to the measurement process defined previously, roughness measurement has to be made along the machined length. In this measurement process, data (xi, zi) of the surface geometry of the piece are obtained. These data need to be recorded in a suitable format so that later they can be used by available software.

A first approach to the study of the surface quality of the mechanized pieces has been made in this work. The arithmetical average roughness, *Ra*, has been selected as a parameter to analyse. According to ISO 4288 standard [15], this parameter is defined as the arithmetical average of the absolute values of the deviations of the profile of roughness *R* and is expressed mathematically by means of the following expression:

(1) $$Ra=\frac{1}{l_{m}}\int_{0}^{l_{m}}|z(x)|dx$$

The data processing consists of calculating and plotting the average value of *Ra* measured on the two or three lines mentioned before.

### 2.8. Statistical Analysis

**Figure 2.**
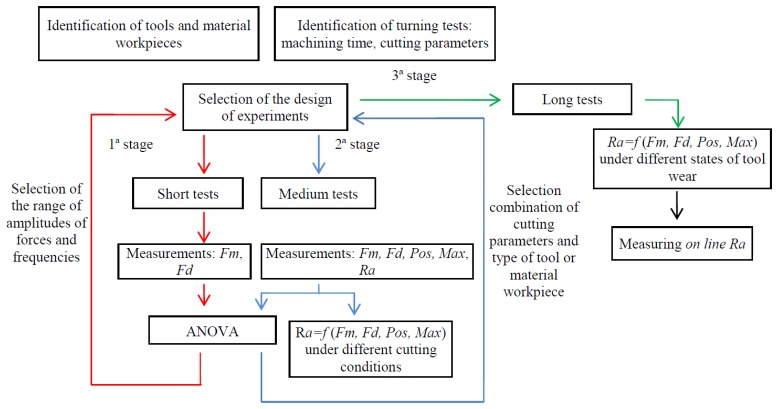
Schematic diagram of the proposed methodology.

For analysing the results of the experimental designs by statistical tests an analysis of variance (ANOVA) is developed. Besides Pareto charts, cube and boxes and whiskers plots are used. Two predictive models of surface roughness, one, under different cutting conditions and, another, under different tool wear states in function of the statistical parameters of forces, are made using a multivariable regression technique. The main stages of the proposed methodology are represented in the following schematic diagram (Figure 2).

## 3. Experimental Application

The turning tests were carried out on workpieces of UNS A97075 aluminium alloy using two types of tools. Concretely, the tools were from the manufacturer SECO (Fagersta, Sweden), namely DCMT11T304-F2 and DCMT11T308-F2 tools with a radius nose of 0.4 mm (R04) and 0.8 mm (R08), respectively, and they were conducted on the Pinacho Modelo L-1/200 lathe (Monzón, Spain).

The force sensor employed was a Kistler 9257B dynamometer (Lorch, Germany) to measure cutting forces in three mutually perpendicular directions corresponding to the *x* direction (feed force *F_f_*), *y* direction (thrust force *F_t_*), and *z* direction (cutting force *F_c_*), which are connected to the Kistler multichannel charge amplifier type 5070A. The values of the selected inputs sensitivities were −7.4 −7.5 −3.7 pC/N, respectively in the directions of *F_x_*, *F_y_* y *F_z_*, and the outputs sensitivities were of 40 N/V in the directions of *F_x_* y *F_y_*, and 80 N/V in the direction of *F_z_*. Force measurements were set at the sample rate of 15 kHz.

The measured numerical values and graphics were stored in a computer by a data acquisition system Dasylab (Amherst, NH, USA). This software was also employed for the monitoring of the turning process. The measurements of the surface roughness, in terms of *Ra*, were made with a Mitutoyo (Kawasaki, Japan) roughness tester and its software Surftest SJ-401.

For the first stage (short tests) the design of experiments selected was a 2^4^ full factorial design with two replications (32 experiments). The workpiece used for these tests was a bar with a diameter of 60 mm and a length of 120 mm.

Afterwards, for the second stage (medium tests) the design of experiments selected was a 2^5^ carried out in two blocks (32 experiments) according to the length of workpiece where the turning process starts. In this case, the number of experiments is not the same that the number of the turning tests which are 16 tests. This is because for each turning pass the workpiece was divided into two machined lengths were the surface roughness measurements were taken and consequently, one turning test corresponds to two experiments. In order to maintain the same conditions from the point of view of the value of diameter of the workpiece machined, the turning tests were carried out on two workpieces with a diameter of 60 mm and a length of 150 mm. The tests were carried out along the 100 mm of their length, so the machined lengths of L1 and L2 employed for surface roughness measurements were 50 mm.

For the last stage, the combination of cutting conditions selected was: feed rate of 0.1 mm/rev, spindle speed of 1,470 rpm, depth of cut of 0.25 mm and the type of tool with radius nose of 0.4 mm (R04). These tests were carried out in four blocks corresponding to four tool states: fresh tool (W0), wear state 1 (W1), wear state 2 (W2) and wear state 3 (W3). To achieve the tool wear on different levels, turning tests were performed with a harder material workpiece and more aggressive cutting conditions during approximately 15 min between one state and another. In each state, with the selected combination of cutting conditions, turning tests were carried out during 15 min. Each 3 min of turning, cutting forces and surfaces measurements were recorded. This time of machining corresponds to three passes on the workpiece along 100 mm of its length.

Finally, to validate the model obtained, additionally, 24 turning tests were performed at same cutting conditions at three levels of wear, situated approximately between the states of tool wear defined in this study.

## 4. Results

The results obtained in the three experimental phases are presented below:

### 4.1. First Stage

Tables 1, 2 and 3 show the analysis of ANOVA of the three components of the arithmetic values for forces, *Fm_z_*, *Fm_x_* and *Fm_z_*, respectively, obtained in the short tests, in which uniquely the influential factors are collected. The rightmost columns of the tables show the percent contribution (*P*) of the factors that were found to be statistically influential on the corresponding response variable. The *p*-values of these factors obtained in the statistical test were lower than 0.05.

At a first glance, it can be observed that *Fm_z_* was affected by a larger number of factors. These were, in order of importance, the feed rate, *f*, the depth of cut, *d*, the type of tool, *T*, the interaction between the spindle speed and feed rate, *N*×*f*, the spindle speed and the interaction of tool and feed rate, *T*×*f*.

**Figure 3.**
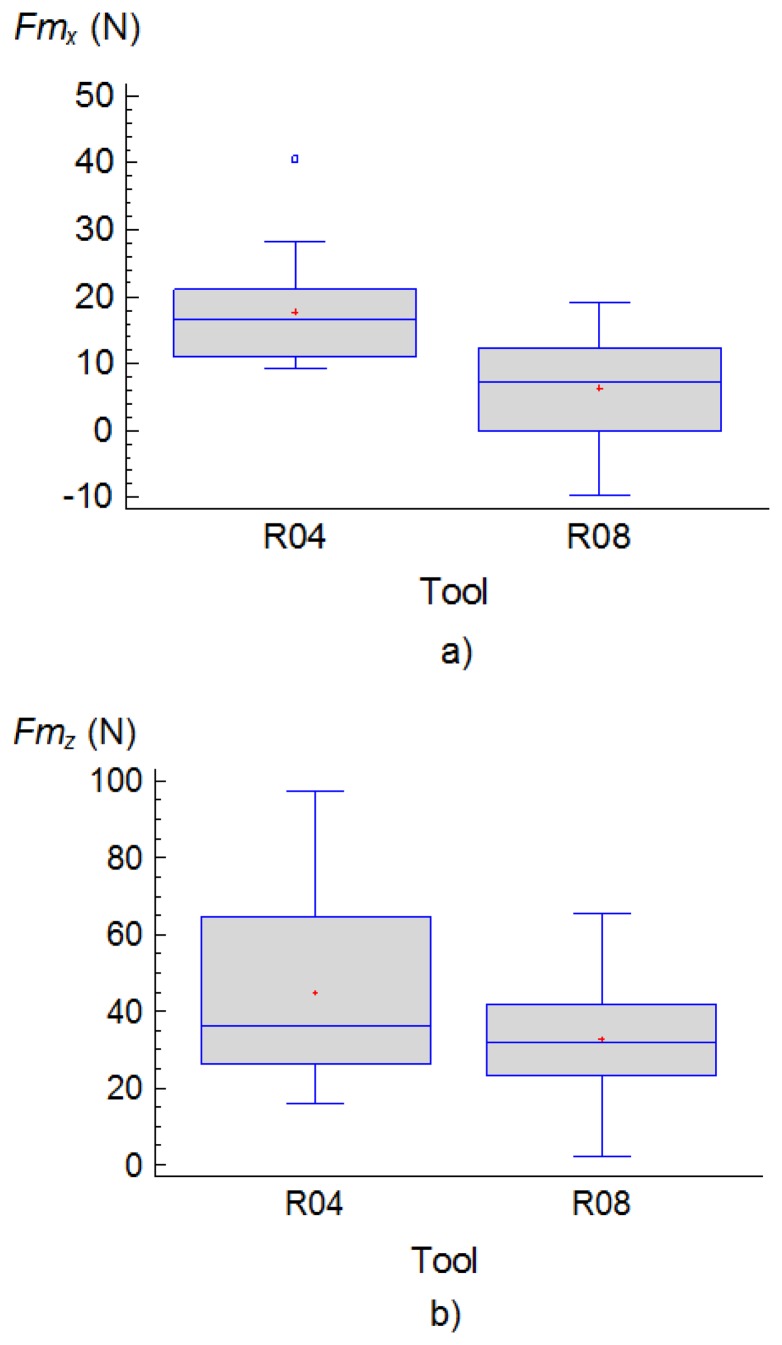
Box and whiskers plots for: (**a**) *Fm_x_* and (**b**) *Fm_z_ versus* the type of tool employed.

**Figure 4.**
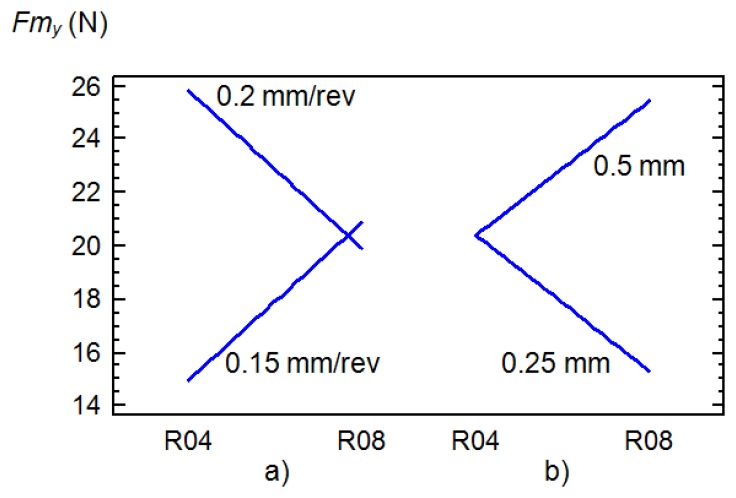
Charts showing the influential interactions for *Fm_y_*: (**a**) tool and feed rate (**b**) tool and cutting depth.

**Figure 5.**
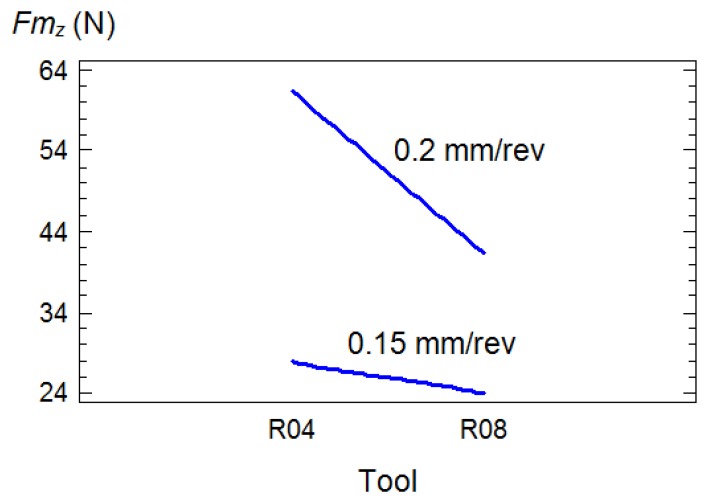
Charts showing the influential interactions for *Fm_z_*: tool and feed rate.

In general, the most important factors were the type of tool, *T*, the feed rate *f* and depth of cut, *d*. Regarding the type of tool, *T*, it was determined that with the use of R04 tools larger values of both components, *Fm_x_* and *Fm_z_*, were generated during the turning process as can be seen in the following charts (Figure 3):

The influence from interaction with other factors on the *Fm_y_* and *Fm_z_* components is also noteworthy. These were the interactions with the feed, *T*×*f* and with the depth of cut, *T*×*d*. With R04 tools, the application of feeds of 0.15 mm/rev led to values obtained for thrust and cutting forces that were significantly lower than with feeds of 0.20 mm/rev (Figure 4), nonetheless with R08 tools, similar values of *Fm_z_* were obtained (Figure 5). In relation with the depth of cut, this factor was sensitive to the thrust forces only in the case of the use of R08 tools (Figure 4).

**Figure 6.**
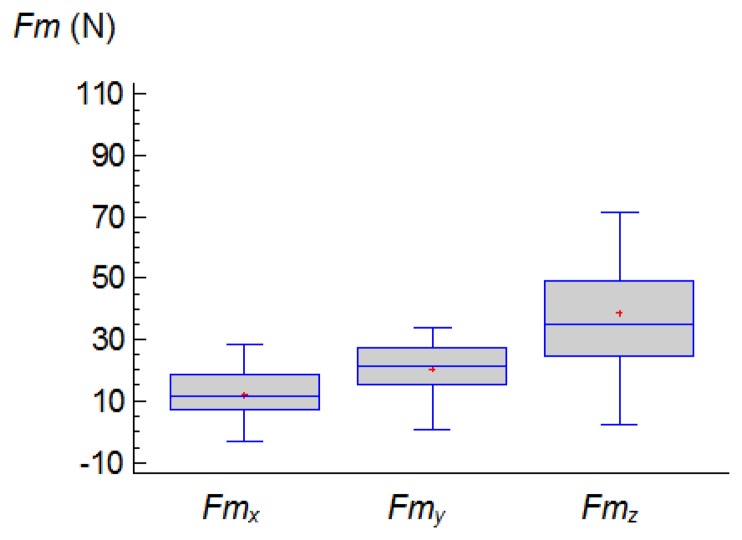
Box and whiskers plot for the three components of the forces: *Fm_x_*, *Fm_y_* and *Fm_z_*.

In relation to the order of magnitude of the forces, the component, *Fm_z_*, reached the greatest values. Figure 6 shows the range of values of the three components obtained, *Fm*_x_, *Fm*_y_ y *Fm*_z_.

So, taking into account these preliminary results, which show the major percent of feed rate influence on the cutting forces, *Fm_z_*, lower values of this cutting parameter were selected for the next experimental phase.

### 4.2. Second Stage

The results of ANOVA analysis of *Ra* obtained during the 2^nd^ experimental phase (medium tests) are presented in the Table 4. The influential factors were the feed rate *f*, the type of tool *T*, the interaction between feed rate and the type of tool *f*×*T*, the interaction between feed rate and the spindle speed *f*×*N* and the interaction between the type of tool and spindle speed, *T*×*N*. Neither of the other two factors, the depth of cut, *d*, and the machined length, *L*, nor their interactions with the other factors contributed to the surface roughness measured.

In relation to the influence of the depth of cut on the surface roughness, opposite results have been reported, therefore, this could indicate that its influence on *Ra* is conditioned by other factors such as the material of the workpiece. In this sense, Huang and Chen [16] developed a predictive model of surface roughness obtained on pieces of aluminium UNS A96061 based on different cutting parameters in which the depth of cut was not included. Nevertheless, the study developed by Rubio and partners [17] in which the surface quality was analysed on aluminium UNS A92024 workpieces, points out that the greater the depth of cut was, the more improved surface quality of the pieces was approached. This effect is attributed to the material adhered to the tool. In the present study, although aluminium UNS A97075 pieces are employed which have also a high tendency to adhere to the tool; the use of much lower depth cut values than the ones employed in the mentioned study has made the influence of such cutting parameter on *Ra* almost negligible.

**Figure 7.**
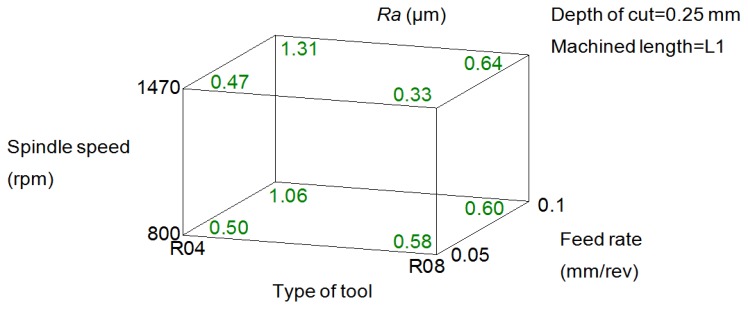
Cube plot of *Ra versus* the feed rate, spindle speed and type of tool.

Figure 7 shows a cube representing the eight combinations of the influential factors on *Ra*: feed rate, tool and spindle speed. It can be seen, as was expected, that the increase of feed rate and the type of tool (radius nose tool) has resulted in a detriment of the surface quality.

Deeper analysis reveals that with both tools R04 and R08, at lower feeds of 0.05 mm/rev, the use of greater values of spindle speeds (1,470 rpm) lower values of *Ra* were obtained. At higher feeds of 0.1 mm/rev this tendency was inverted, achieving a better quality surface with the application of spindle speeds of 800 rpm.

It is also important to remark that with the use of R04 tool for certain combination of the feed rate and spindle speed, this is, 0.05 mm/rev and 800 rpm, respectively, a lower value of *Ra* (0.51 μm) was obtained than with R08 tool (0.58 μm).

The selection of a combination of cutting condition for the following phase was made based on the range of values of *Ra*, usually specified for stressed parts in which close fits are required (ASME B46.1 [18]). These are values between 0.8 and 1.6 μm. So that the combination of cutting condition was selected that led to obtain the highest value of *Ra* according to the Figure 7: 1.31 μm. The reason was, as mentioned previously, that it is expected that the material adhered to the tool improves the surface quality; a fact has been reported in different studies. Therefore, the cutting conditions selected for the 3rd experimental phase were: R04 tool, feed rate of 0.1 mm/rev and spindle speed of 1,470 rpm.

Additionally, in this experimental phase, a linear multivariable predictive model of surface roughness based on the cutting forces under different cutting conditions was obtained (Equation (2)):

(2) $$[Ra=0.927405-0.203466*Fd_{x}+0.532696*{Max}_{x}*Fd_{x}-0.434305*{Max}_{\text{y}}-\;0.00155221*Fm_{\text{x}}*Fm_{\text{y}}-0.00209439*Pos_{\text{x}}*{Max}_{x}+\;0.000573404*Fm_{\text{z}}*Fm_{\text{z}}$$

According to the *R*-square value this model explains a 93.71% of variability on *Ra*.

### 4.3. Third Stage

**Figure 8.**
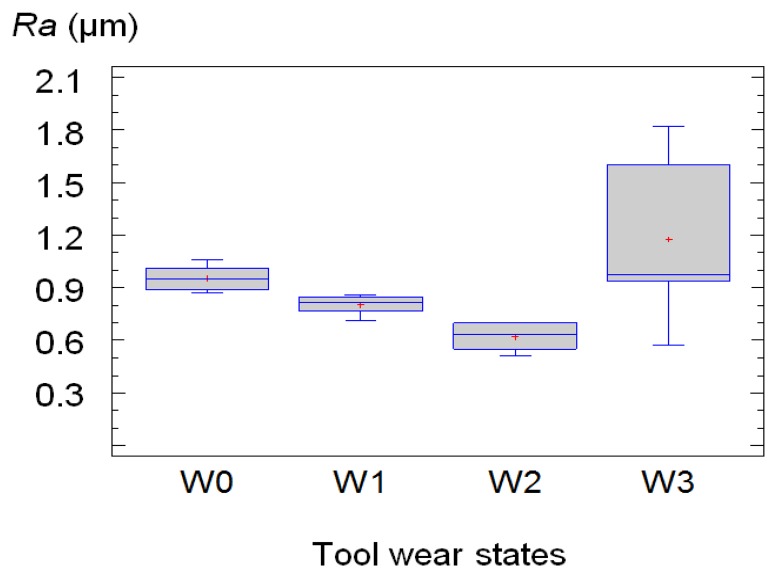
Boxes and whiskers plot of *Ra* for different levels of tool wear: Fresh tool (W0), wear state 1 (W1), wear state 2 (W2) and wear state 3 (W3).

In Figure 8 the values of *Ra* obtained from this experimental phase (long tests) *versus* the different levels of tool wear defined in the previous section, W0, W1, W2 y W3, are plotted:

It can be seen in Figure 8 that the values of *Ra* decreased with the time of machining until a certain level of wear of the tool, in this study, that was until the state 2; during the machining process, the material adhered to the tool provokes the growth of the tool nose radius and, consequently, the surface quality improves, at least within a certain time.

Also in this experimental phase, a model of surface roughness was developed based on the parameters of the cutting forces; in this case, under the same cutting conditions that were previously selected (R04 tool, feed rate of 0.1 mm/rev and spindle speed of 1,470 rpm). The model obtained is described by the following potential function (Equation (3)):

(3) $$\left[Ra=Fd_{x}^{-2,44208}*{Max}_{y}^{-2,22136}*Fd_{z}^{1,21826}*{\left(Fd_{y}*Pos_{z}\right)}^{0,0726374}\right]$$

The *R*-square value for this model was 67.4%, considerably lower than the 93.7% one obtained in the previous experimental phase. The adjustment of the different independent variables was made in a lesser extent due to the fact that the tests were carried out under the same cutting conditions. Therefore, less difference between the values of the cutting forces was achieved and consequently the surface roughness was less sensitive to the variations of these values.

**Figure 9.**
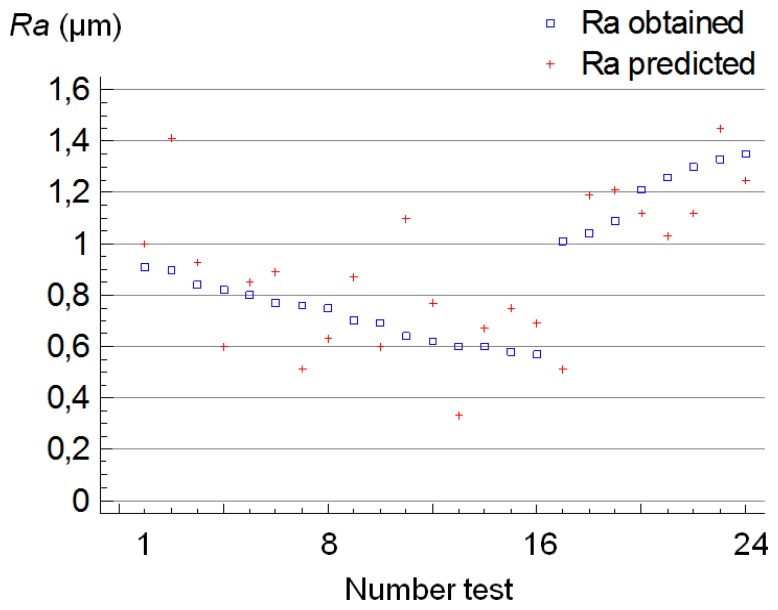
Plot with the values of *Ra* for the validation of the model.

Afterwards, for the validation of this model, other turning tests were performed, under the same conditions, recording eight surface roughness and eight cutting forces measurements at three levels of tool wear situated, approximately, between the wear levels defined. These were between the states of wear of, W0 and W1, W1 and W2, and, W2 and W3. In Figure 9 the values of *Ra* measured and the values predicted by the model are plotted.

It can be observed that the deviations between both mentioned values obtained in seven tests (number 2, 4, 7, 11, 13, 17 and 21) were higher than 0.2 μm. For such tests, it could be considered that the adjustment of the model is not sufficiently accurate, so that in terms of percent, the result of this validation reveals that the model was effective for, approximately, 70% of the values of *Ra* obtained.

## 5. Conclusions

A methodology has been developed for the evaluation of the effect that tool wear has on the surface roughness obtained by a turning process. Firstly, the most improved cutting parameters are selected for some certain surface roughness requirements and secondly, the evaluation of the surface roughness has been carried out in real time through the cutting forces. Concretely, a model of surface roughness is developed under the same cutting parameters with the objective to detect when the level of tool wear is high enough to deteriorate the surface quality of the workpiece. In this aspect, the proposed model is novel with respect to those which have been reported in previous studies. This is a very important challenge, so it would lead to increased tool life by selecting the right moment for its replacement.

Furthermore, the use of this methodology makes it possible to carry out comparative analyses between different experimental studies towards to the prediction of surface roughness in turning processes.

Following this trend, different protocols were designed in which the following have been included: the definition and identification of turning process (cutting parameters, machining time, state of the tool wear), the identification of the type of tools, the experimental planning (design of experiments) and the procedures for the turning tests and for the measuring of both the cutting forces and the surface roughness.

In this study, the proposed methodology was applied for the development of a predictive model of surface roughness of pieces of UNS A97075 aluminium alloy based on statistic parameters calculated from the three cutting force components (feed, thrust and cutting forces), in the time and frequency domain under different cutting parameters (feed rate, spindle speed, depth of cut) and different types of tools (tools with 0.4 and 0.8 mm radius). In a preliminary step, the most improved cutting conditions for the surface roughness quality commonly required in the aeronautical sector were obtained. With such cutting conditions, another predictive model of surface roughness based on cutting forces was obtained under different tool wear states that simulate the behaviour of the tool, in terms of surface roughness, throughout its life. The validation of this model reveals that was effective for, approximately, 70% of the values of *Ra* obtained.

Taking to account the percent of the model that could not be explained, in order to improve the adjustment of the model for further studies, it is recommended to analyse a wider range of frequencies in which forces perform, as well as the implementation of signals from other sensors, furthermore the consideration of more levels of wear of the tool and to carry out a larger number of machining tests.

## Figures and Tables

**Table 1.** ANOVA analysis of *Fm_x_*.

**Source**	**Sum of Square**	**DF**	***F* value**	***p* value**	**Contribution (%)**
*T*	1,018.31	1	21.33	0.0001	33.6
*d*	494.677	1	10.36	0.0033	16.3
*F*×*N*	224.095	1	4.69	0.0392	7.4
Blocks	13.5148	1	0.28	0.5990	
Error	1,288.76	27			
Total	3,039.36	31			

**Table 2.** ANOVA analysis of *Fm_y_*.

**Source**	**Sum of Square**	**DF**	**F Value**	**p Value**	**Contribution (%)**
*f*	200.145	1	4.60	0.0414	9.3
*d*	206.101	1	4.74	0.0387	9.6
*T*×*f*	277.484	1	6.38	0.0180	12.9
*T*×*d*	204.965	1	4.71	0.0392	9.6
Blocks	137.171	1	3.15	0.0874	
Error	1,130.5	26			
Total	2,156.36	31			

**Table 3.** ANOVA analysis of *Fm_z_*.

**Source**	**Sum of Square**	**DF**	***F* Value**	***p* Value**	**Contribution (%)**
*T*	1,134.73	1	17.13	0.0004	8.9
*f*	5,164.25	1	77.95	0.0000	40.1
*N*	8,17.343	1	12.34	0.0000	6.4
*d*	2,541.26	1	38.36	0.0000	19.8
*T*×*f*	531.241	1	8.02	0.0092	4.2
*f*×*N*	1,099.21	1	16.59	0.0004	8.6
Blocks	0.452914	1	0.01	0.9348	
Error	1,590.04	24			
Total	12,878.5	31			

**Table 4.** ANOVA analysis of *Ra*.

**Source**	**Sum of Square**	**DF**	***F* Value**	***p* Value**	**Contribution (%)**
*T*	0.72	1	77.22	0.0000	23.84
*f*	1.47061	1	157.63	0.0000	48.66
*f*×*T*	0.5618	1	60.26	0.0000	18.60
*T*×*N*	0.0946125	1	10.15	0.0037	3.13
*f*×*N*	0.16245	1	17.42	0.0003	5.38
Blocks	0.00015	1	1.25	0.4235	
Residual	0.231162	24			
Total	3.3202405	31			
